# Anatomical Retinal Changes after Photodynamic Therapy in Chronic Central Serous Chorioretinopathy

**DOI:** 10.1155/2018/4081874

**Published:** 2018-02-06

**Authors:** María Pilar Ruiz-del-Tiempo, Pilar Calvo, Antonio Ferreras, Jesús Leciñena, Luis Pablo, Oscar Ruiz-Moreno

**Affiliations:** ^1^Department of Ophthalmology, Gregorio Marañón University General Hospital, Madrid, Spain; ^2^IIS-Aragón, Department of Ophthalmology, Miguel Servet University Hospital, Zaragoza, Spain; ^3^University of Zaragoza, Zaragoza, Spain

## Abstract

**Purpose:**

To evaluate anatomical retinal changes measured by spectral-domain optical coherence tomography (SD-OCT), after applying photodynamic therapy (PDT) for treatment of chronic central serous chorioretinopathy (CSC).

**Methods:**

A retrospective analysis was conducted on 43 patients (48 eyes) with chronic CSC treated with PDT. Visual acuity (VA), central retinal thickness (CRT), outer nuclear layer (ONL) thickness, subretinal fluid (SRF), and photoreceptor ellipsoid zone (EZ) measured by SD-OCT were collected at baseline and at 3, 6, and 12 months after PDT. Differences between normally distributed variables were calculated by a paired-sample *t*-test; *p* < 0.05 was considered statistically significant.

**Results:**

Mean age was 50 ± 9.8 years. Mean time from diagnosis to PDT was 12.5 months. Baseline VA was 0.51 ± 0.24 and significantly improved (*p* < 0.001) to 0.74 ± 0.26 one year after PDT. CRT and SRF significantly decreased (*p* < 0.001) at 3, 6, and 12 months after treatment. ONL thickness and EZ did not significantly change at any point during follow-up.

**Conclusions:**

Not significant changes were found in the ONL or EZ 12 months after PDT.

## 1. Introduction

Central serous chorioretinopathy (CSC) is characterized by the development of one or more well-circumscribed, neurosensorial retinal detachments commonly associated with serous pigment epithelial detachments (PEDs) and tends to affect the retina at the macular area. The pathophysiology is not completely understood, but the hallmarks of this condition include choroidal hyperpermeability and deficient pumping functions at the level of the retinal pigment epithelium (RPE) [1, 2].

CSC is the fourth most frequent retinal pathology after age-related macular degeneration (AMD), diabetic retinopathy, and retinal vein occlusion [3, 4]. It occurs primarily in healthy men between 25 and 55 years of age [5]. Symptoms included decreased visual acuity (VA) and color vision, micropsia, metamorphopsia, or paracentral scotoma. It is related to type A personality, stress, and patients with an elevated level of corticosteroids [6].

The natural course of acute CSC is usually excellent; most patients (80–90%) undergo spontaneous resolution of subretinal fluid (SRF) within six months. However, some patients suffer from chronic CSC, causing permanently diminished VA, and many (40–50%) experience one or more recurrences. Verteporfin (Visudyne; Novartis, Basel, Switzerland) ocular photodynamic therapy (PDT) has been proven off-label as an effective treatment to resolve chronic CSC, improving VA and reabsorbing the SRF [7].

PDT has been widely used in treating wet AMD [8–10]. Serious ocular adverse events are uncommon and include decrease in vision, secondary choroidal neovascularization (CNV), RPE tears, and choroidal nonperfusion. Systemic photosensitivity reactions have been reported as well, usually occurring within 24 hours of verteporfin infusion. Patients are advised to avoid sun exposure and use protective clothing for the first 2 days after treatment.

The purpose of the present study was to evaluate anatomical retinal changes measured by spectral-domain optical coherence tomography (SD-OCT), after applying PDT for treatment of chronic CSC.

## 2. Methods

We retrospectively identified 75 patients with chronic CSC treated with PDT. All patients were examined at the Department of Ophthalmology, Miguel Servet University Hospital. The design of the study followed the tenets of the Declaration of Helsinki, and the protocol was approved by the Clinical Research Ethics Committee of Aragon.

Subjects were eligible if they were adults with a diagnosis of chronic CSC (defined as persistence of SRF for more than 3 months) with a follow-up of at least 12 months. Exclusion criteria included opacity of the optical media that could interfere with the quality of the SD-OCT; pre-existing retinal, choroidal, or optic nerve pathology; and previous ocular treatment with focal laser or intravitreal agents.

A full ophthalmologic examination was performed in all patients including clinical history, best-corrected visual acuity (BCVA, decimal scale), examination of the anterior segment using a slit lamp, Goldmann applanation tonometry, ophthalmoscopy of the posterior segment, intravenous fluorescein angiography, and SD-OCT.

All patients received full standard dose of verteporfin (6 mg/m^2^), which was infused over 10 minutes, followed by laser treatment 5 minutes after. Laser was applied to the area of hyperpermeability that was detected during IVFA. The total light energy was 50 J/cm^2^ and irradiance of 600 mW/cm^2^ of 689 nm light over 83 s.

All eyes were scanned by the SD Cirrus OCT system (Carl Zeiss Meditec, Dublin, California; macular cube 512 × 128 scan and HD 5-line raster; software version 6.2) after mydriasis (0.5% tropicamide; Alcon Laboratories Inc., Fort Worth, Texas). All scans had signal strength of at least 7.

Full thickness of the outer nuclear layer (ONL), photoreceptor ellipsoid zone (EZ; distance between the lower border of the external limiting membrane and the anterior surface of the RPE), SRF, and central retinal thickness (CRT) were manually drawn at the subfoveal point by 2 retinal specialists (J.L. and O.R.) who were blind to all clinical information using the built-in caliper function of the Cirrus OCT. To obtain the anatomical measurements, the HD 5-line raster scan was used. It consists of 4096 A-scans in each of the five lines, centered on the fovea. CRT was obtained manually through measuring the distance between the inner surface of the RPE and the outer surface of the neurosensory retina on the horizontal HD 5-line raster scan centered on the fovea.

### 2.1. Statistical Analysis

Statistical analysis was performed using IBM's statistical software (SPSS version 22.0; IBM Corporation, Somers, New York). Descriptive findings were reported as mean ± SD. All study variables were normally distributed as verified by the Kolmogorov-Smirnov test (K-S of 1 sample). Differences between normally distributed variables were calculated by a paired-sample *t*-test; *p* < .05 was considered statistically significant.

To analyse the relationship between quantitative variables (BCVA, disease duration, thickness measurements, etc.), the Pearson correlation was used.

## 3. Results

48 eyes of 43 chronic CSC patients were finally included. Among the patients, 12 (28%) were women and 31 (72%) were men, with a mean age of 50 ± 9.8 years (28%). Mean duration of CSC prior to treatment was 7.6 ± 2.7 months. Mean period of follow-up after PDT was 12.5 months. The mean PDT spot area was 24.2 ± 10.6 mm^2^. Mean baseline BCVA was 0.51 (20/40) ± 0.24, and mean baseline CRT was 313.6 ± 86.6 *μ*m. Clinical characteristics are shown in Table 1.

**Figure 1:**
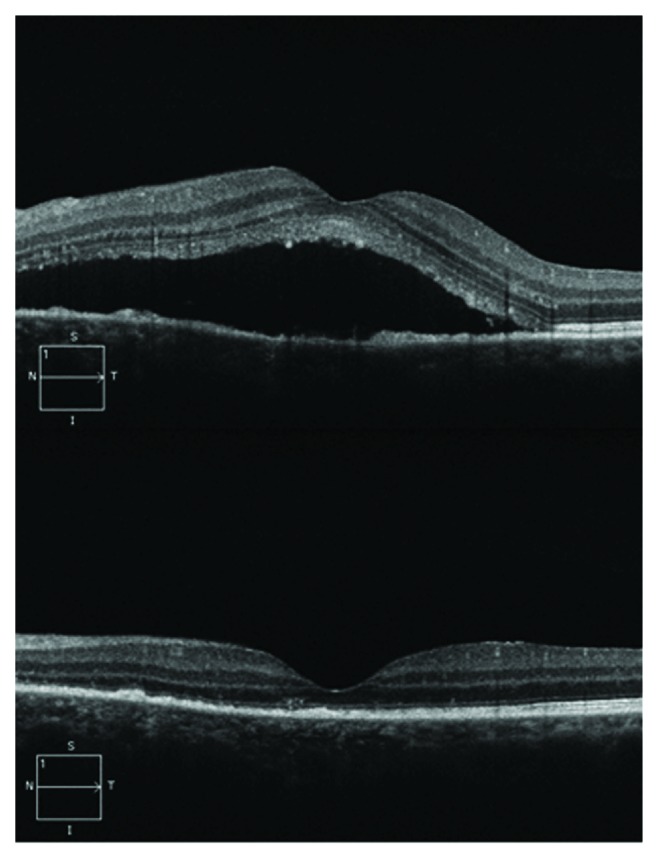
SD-OCT showing SRF resolution after PDT treatment.

After initial PDT, absorption of SRF (Figure 1) was complete in 39 eyes (81.2%); six more eyes (12.5%) resolved after additional PDT. Two eyes (4.2%) did not show response after third PDT, and one eye (2.1%) was not treated because the patient refused further treatment. No patients developed systemic or ocular adverse events related to PDT.

### 3.1. SD-OCT Changes

Mean CRT at baseline and at 3, 6, and 12 months after PDT was 313.63 ± 86.63, 183.79, 189.44, and 177.59, respectively. ONL, SRF, and EZ thicknesses are summarized in Table 2. After PDT, CRT and SRF became significantly thinner at 3, 6, and 12 months (all *p* < 0.001). ONL and EZ did not show significant differences during the follow-up.

The mean BCVA at baseline and at 3, 6, and 12 months after PDT was 0.51 (20/40) ± 0.24, 0.7 ± 0.3, 0.67 (20/30) ± 0.24, and 0.74 (20/25) ± 0.26, respectively. Baseline BCVA significantly improved at 3, 6, and 12 months after PDT (all *p* < 0.001).

A strong correlation was found between visual acuity (VA) at baseline, 3 months (*R* = 0.747; *p* = 0.001), and 6 months (*R* = 0.720; *p* = 0.001). Final VA (12 months) was inversely related with disease duration (*R* = −0.397; *p* = 0.015). A negative relation was also found between disease duration and ONL thickness at 12 months after PDT (*R* = −0.415; *p* = 0.011).

## 4. Discussion

PDT is an effective treatment to resolve chronic CSC, achieving anatomical response in almost 96% of the cases and improving VA in 75%. Similar results have been previously published [7, 11–14]. Our study showed that after initial PDT, absorption of SRF was complete in 39 eyes (81.2%). Fujita et al. [15] studied 204 eyes with chronic CSC and reported 182 eyes (89%) with resolution of SRF at 12 months after half-dose PDT. Regarding VA, our study found a significant increase in vision after PDT treatment. Moreover, the obtained data indicated a negative correlation of final VA (12 months) with disease duration (*R* = −0.397; *p* = 0.015). Additionally, a positive correlation was found between baseline VA and at 3 months (*R* = 0.747; *p* = 0.001) and 6 months (*R* = 0.720; *p* = 0.001).

To our knowledge, this is the first study measuring specific retinal layers after using full-fluence PDT in chronic CSC. Although some authors have reported ocular complications such as choroidal ischemia, RPE atrophy, or secondary CNV using conventional dosage of PDT [16], its use is widely extended in daily practice and is considered a safe procedure. 12 months after PDT, we did not find significant changes at the level of the ONL thickness or EZ measured with SD-OCT. CSC primarily alters the outer segment. Thinning of the ONL and poor VA at baseline have been correlated with worse outcomes [17, 18]. Since mean disease duration was relatively short in this sample (1.5 years), we hypothesized that longer disease duration also matched with these two features.

Silva et al. [19] evaluated in 2013 the safety and efficacy of PDT at 48 months in 42 chronic CSC patients (46 eyes). Stratus 3 OCT (time-domain) was performed to measure SRF, neural retina thickness (NRT), and CRT. They found a complete resolution of SRF in 93.4% of the eyes and a significant decrease of CRT, but NRT remained stable during the 48 months of follow-up. The retinal atrophy was associated with the disease itself but not related to PDT treatment. They also reported a significant improvement in vision in 74% of the eyes.

Vasconcelos et al. [20] also published the anatomical changes 5 years after standard PDT for chronic CSC. They included 15 patients (17 eyes), and they also used Stratus OCT. They also concluded that the morphological and functional chorioretinal changes observed 5 years after PDT were more related to the chronic disease than with the treatment provided. Their CRT significantly decreased, but their NRT remained stable.

Using SD-OCT, 4 years later, we were able to measure specific layers, and our findings showed that the mean ONL thickness did not differ significantly from that before PDT; however, ONL thickness was inversely related with disease duration (*R* = −0.415; *p* = 0.011).

Matsumoto et al. [21] investigated the ONL thickness and EZ in 20 CSC eyes and 10 controls on Fourier domain (FD) OCT. They found elongation of photoreceptor outer segments and decreased ONL thickness in CSC (without any treatment). Significant thinning of the ONL (median, 75 *μ*m) compared with that of the normal eyes (median, 153 *μ*m) suggests that apoptosis of the photoreceptors occurs in the detached retina. Our findings support that PDT did not significantly change ONL thickness. It is already diminished because of the disease itself.

Regarding EZ, there is conflicting evidence about the influence of PDT on photoreceptors. Previous histopathological studies reported that 1 week after PDT, Schlötzer-Schrehardt et al. [22] did not find RPE or neurosensory retina damage. On the other hand, recent OCT studies [23] have suggested that a temporal change of photoreceptors was found in patients treated with PDT, but their measurements were taken outside the fovea.

This retrospective study has several limitations. The sample size was relatively small, and the follow-up period (12 months) was relatively short. We did not evaluate additional useful functional retina tests as visual field or multifocal electroretinography. Choroidal thickness (CT) was not evaluated because data regarding axial length and refraction were not available. OCT scans were performed at different times in each patient, which also affects CT. Treatment location was not registered, and retinal measurements were only made at the subfoveal area. There is the need for further studies to ascertain the relationship between changes in ONL layer and EZ and the long-term risk of retinal toxicity with PDT treatment.

In conclusion, this study indicated that ONL and EZ did not decrease thickness in chronic CSC patients 12 months after PDT therapy, using SD-OCT.

## Figures and Tables

**Table 1:** Clinical characteristics of the studied sample.

	Mean	SD
Age (years)	50.31	9.81
Male, *n* (%)	31 (72%)	NA
PDT spot area (mm^2^)	24.28	10.66
BCVA (Snellen)	0.51 (20/40)	0.24
CRT (*μ*m)	313.63	86.63

PDT: photodynamic therapy; BCVA: best-corrected visual acuity; CRT: central retinal thickness.

**Table 2:** Comparison of SD-OCT measurements after PDT treatment.

	Baseline	3 months	6 months	12 months
CRT (*μ*m) A	313.63 ± 86.63	183.79 ± 47.05^∗^	189.44 ± 51.67^∗^	177.59 ± 29.65^∗^
ONL (*μ*m) B	81.43 ± 17.26	85.94 ± 18.63	80.56 ± 19.81	83.68 ± 20.87
SRF (*μ*m) C	151.74 ± 87.15	8.49 ± 37.15^∗^	20.87 ± 55.26^∗^	1.61 ± 9.67^∗^
EZ (*μ*m) D	81.33 ± 36.88	77.31 ± 28.11	85.71 ± 12.7	81.67 ± 28.01

^∗^ *P* < 0.001. CRT: central retinal thickness; ONL: outer nuclear layer; SRF: subretinal fluid; EZ: ellipsoid zone.
